# Mediating and Moderating Effects of Iron Homeostasis Alterations on Fetal Alcohol-Related Growth and Neurobehavioral Deficits

**DOI:** 10.3390/nu14204432

**Published:** 2022-10-21

**Authors:** R. Colin Carter, Neil C. Dodge, Christopher D. Molteno, Ernesta M. Meintjes, Joseph L. Jacobson, Sandra W. Jacobson

**Affiliations:** 1 Departments of Emergency Medicine and Pediatrics, Institute of Human Nutrition, Columbia University Vagelos College of Physicians and Surgeons, New York, NY 10032, USA; 2 Department of Human Biology, University of Cape Town Faculty of Health Sciences, Cape Town 7925, South Africa; 3 Department of Psychiatry and Behavioral Neurosciences, Wayne State University School of Medicine, Detroit, MI 48201, USA; 4 Department of Psychiatry and Mental Health, Faculty of Health Sciences, University of Cape Town, Cape Town 7925, South Africa

**Keywords:** fetal alcohol syndrome (FAS), fetal alcohol spectrum disorders (FASD), growth restriction, iron, iron deficiency, iron deficiency anemia, prenatal alcohol exposure, recognition memory, symbolic play, temperament

## Abstract

We have previously demonstrated prenatal alcohol exposure (PAE)-related alterations in maternal and infant iron homeostasis. Given that early iron deficiency and PAE both lead to growth restriction and deficits in recognition memory and processing speed, we hypothesized that PAE-related iron homeostasis alterations may mediate and/or moderate effects of PAE on growth and neurobehavior. We examined this hypothesis in a prenatally recruited, prospective longitudinal birth cohort [87 mother-infant pairs with heavy prenatal alcohol exposure (mean = 7.2 drinks/occasion on 1.4 days/week); 71 controls], with serial growth measures and infant neurobehavioral assessments. PAE was related to growth restriction at 2 weeks and 5 years, and, in infancy, poorer visual recognition memory, slower processing speed, lower complexity of symbolic play, and higher emotionality and shyness on a parental report temperament scale. Lower maternal hemoglobin-to-log(ferritin) ratio, which we have shown to be associated with PAE, appeared to exacerbate PAE-related 2-week head circumference reductions, and elevated maternal ferritin, which we have shown to be associated with PAE, appeared to exacerbate PAE-related visual recognition memory deficits. In causal inference analyses, PAE-related elevations in maternal ferritin and hemoglobin:log(ferritin) appeared to statistically mediate 22.6–82.3% of PAE-related growth restriction. These findings support potential mechanistic roles of iron homeostasis alterations in fetal alcohol spectrum disorders (FASD).

## 1. Introduction

Prenatal alcohol exposure (PAE) is the most common preventable cause of developmental disability worldwide. Fetal alcohol spectrum disorders (FASD) affect 2.0–7.1% of the school age population in the US and Western Europe and 13.6–30.6% in South Africa [1,2,3,4]. FASD are characterized by a range of deficits in neurobehavior and cognition; fetal alcohol syndrome (FAS) and partial FAS (PFAS) are also characterized by specific facial dysmorphology, small head circumference, and/or growth restriction [5,6]. Teratogenic effects of alcohol have been reported in every organ system [6,7,8,9,10].

Both human and animal studies have demonstrated alterations in maternal and infant iron homeostasis induced by PAE. In our prospective, longitudinal birth cohort study in Cape Town, South Africa, infants exposed to prenatal binge drinking were 3.6 times more likely to have iron deficiency anemia (IDA) at 12 months than infant whose mothers did not binge drink [11]. The mechanisms underlying this PAE-related increase in IDA were unclear, as data regarding maternal and fetal iron status were not available. More recently, in a subsequent Cape Town prospective longitudinal birth cohort, we replicated our earlier finding of PAE-related increases in infant IDA and elucidated potential maternal-fetal mechanisms [12]. In the mother, alcohol consumption was associated with elevations in prenatal ferritin and hepcidin, as well as reductions in hemoglobin-to-log(ferritin) ratio, signifying a shift of iron into storage at the expense of other biologic processes such as erythropoiesis and, possibly, placental iron transfer. Indeed, in analyses of placental tissue samples stained for iron transport proteins, PAE was associated with decreases in the ratio of ferroportin-1 to transferrin-receptor-1, which is seen when iron on the maternal side is insufficient to meet the needs of the placenta and fetus [13]. PAE-related elevations in maternal ferritin and reductions in hemoglobin-to-log(ferritin) ratio partially mediated a similar shift of iron into storage at the expense of hematopoeisis in the neonate, as evidenced by PAE-related decreases in hemoglobin-to-log(ferritin) ratio and increases in ferritin at age 2 weeks. PAE-related decreases in neonatal hemoglobin-to-log(ferritin) ratio, in turn, partially mediated the relation of PAE to increased IDA prevalence at 6.5 months. Notably, all of the PAE-related alterations in maternal, placental, and infant iron homeostasis seen were independent of maternal dietary iron intake and use of prenatal iron supplements, which were similar between heavy drinking women and abstainers/light-drinkers. Smith and colleagues demonstrated similar findings in a series of experimental rat models, including PAE-related increases in maternal and fetal anemia and hepcidin, as well as decreased fetal brain iron despite increased iron stores in the fetal liver [14,15,16,17].

Since infant iron deficiency (ID) has been associated with a range of long-term neurodevelopmental deficits [18,19,20], the alterations in iron homeostasis we have found to be related to PAE may have important implications in FASD. Miller and colleagues found that PAE decreased total brain tissue iron despite increases in tissue ferritin [21]. PAE and ID in early infancy are both associated with: growth restriction; poorer recognition memory, information processing speed, and eyeblink conditioning; and higher emotionality and lower activity on temperament measures [9,19,20,22,23,24,25,26,27,28,29,30,31,32]. Furthermore, in our original human birth cohort, PAE-related growth restriction was more severe among children who had had IDA in infancy [7,11]. Similarly, in Smith and colleagues’ rat models [14,17,33], PAE-related infant ID exacerbated teratogenic effects of PAE on growth and behavior. We thus hypothesized that PAE-related alterations in maternal and infant iron homeostasis mediate and/or moderate effects of PAE on growth, neurobehavior, and temperament. We tested these hypotheses in data from our more recent prospective longitudinal birth cohort.

## 2. Materials and Methods

### 2.1. Sample

From 2011–2015 we recruited women from two antenatal clinics in Cape Town, South Africa, to participate in a prospective, longitudinal cohort study examining developmental effects of PAE [34,35]. Alcohol consumption at recruitment was ascertained in timeline follow-back interviews [36,37]. If a woman averaged at least 1.0 oz (30 mL) absolute alcohol (AA)/day (≈1.67 standard drinks) or reported binge drinking (≥2.0 oz (60 mL) AA/drinking occasion), she was invited to participate. Women who abstained or drank only minimally (with no binge episodes) were invited to participate as controls. Maternal exclusion criteria at recruitment included age <18 years, HIV infection, multiple gestation pregnancy, and pharmacologic treatment for medical conditions. Infant exclusion criteria were chosen prior to study initiation due to potential developmental consequences of such conditions; they included major chromosomal anomalies, seizures, neural tube defects, very low birthweight (<1500 g), and extreme prematurity (weeks gestation <32 weeks). We have previously reported that infants with missing data were similar to those seen at 2 weeks and 6.5 months in terms of maternal demographics, alcohol and drug use, weeks gestation at study entry, and maternal dietary iron intake, iron supplement use, ferritin, and hemoglobin [12]. The final sample included 158 mother-infant pairs (87 with heavy PAE, 71 controls). Consents and interviews were conducted in the mother’s preferred language (Afrikaans or English). Women reporting alcohol or drug use were counseled by our staff about the risks of prenatal alcohol and drug use and were encouraged to stop or reduce their alcohol and/or drug use. Women were also offered referrals to substance use treatment programs (South African National Council on Alcoholism and Drug Dependence, Belleville, South Africa).

### 2.2. Ascertainment of Maternal Alcohol, Cigarette Smoking, and Drug Use

At recruitment and 4 and 12 weeks later, women were interviewed about their alcohol consumption on a day-by-day basis during the previous 2 weeks, with recall linked to specific times of daily activities in timeline follow-back interviews [36,37]. Women were also interviewed regarding cigarette and drug use (cocaine, methamphetamine, opiates, methaqualone, and marijuana); drug use reports were validated by urine ELISA drug testing [8]. Three summary alcohol measures were calculated by averaging across pregnancy: oz AA/day, oz AA/drinking occasion, and frequency of drinking (1 oz = 30 mL). Based on prior studies in this cohort [36,38], drinking days/week was used as the PAE predictor variable except where otherwise noted.

### 2.3. Hematological and Biochemical Iron Indices

Venous blood samples were obtained from mothers at recruitment (mean weeks gestation = 25.6; visit 1 in Table 1) and 12 weeks later (mean weeks gestation = 34.0; visit 2 in Table 1) and from infants at 2 weeks and 6.5 months postpartum (corrected for prematurity) and analyzed for complete blood count and serum ferritin. Ferritin is a measure of iron in storage. C-reactive protein and serum soluble transferrin receptor concentrations were assayed on maternal samples and infant samples at 6.5-months. Maternal values from the two visits were averaged. Anemia of inflammation for mothers was defined as normocytic (mean corpuscular volume (MCV) ≥ 80 fL) anemia with ferritin > 30 µg/L [39]. Hemoglobin-to-log(ferritin) ratio was calculated to assess the balance of iron available for erythropoiesis (hemoglobin) vs. iron in storage (ferritin) [12]. Infant ID at age 6.5 months was defined as (1) serum ferritin < 12 µg/L or (2) in cases with CRP > 5 mg/L, elevated sTfR (>8.5 mg/L for mothers; >8.3 mg/L at 6.5 months) [40,41,42]. Anemia was defined as hemoglobin < 11.0 g/dL [42]. Iron deficiency anemia was defined as the presence of both iron deficiency and anemia.

### 2.4. Demographics and Control Variables

Women were interviewed at prenatal visits regarding their demographic background, including age, gravidity, and education. Weeks gestation at delivery was calculated based on early pregnancy ultrasound; when this was not available, last menstrual period was used. Infant sex and birthweight were obtained from medical records.

### 2.5. Infant Anthropometric Outcomes

Weight, length, and head circumference were measured at ages 2 weeks and 5 years by research assistants (trained by RCC) using standard WHO protocols [43]. Age/sex-specific *z*-scores were calculated for length/height, weight, at both ages and head circumference at 2 weeks using WHO norms. Raw values (cm) were used for analyses of head circumference at age 5 years since WHO reference norms are not available for this measure at that age.

### 2.6. Infant Neurobehavioral and Temperament Outcomes

Visual recognition memory, and processing time, an inverse measure of processing speed, were assessed at 6.5 months using the Fagan Test of Infant Intelligence [44]. In this assessment, the infant is seated on the mother’s lap and shown two identical photos, followed by a novel photo paired with the familiar one. At this age, the normative response is to look at the novel stimulus for a longer time period, which indicates that the infant is able to recall the familiar stimulus and discriminate it from the novel stimulus. Infant fixation time was recorded on a computer, and visual recognition memory, assessed as percent novelty preference, was computed by dividing duration of time looking at the novel stimulus by total time looking at the paired familiar and novel stimuli for each of 10 novel stimuli problems. In addition, as described in Colombo et al. [45], mean fixation duration (processing time) was computed for each problem by dividing the total duration looking time by the number of looks. A pattern of shorter looks reflects faster processing of information and has been found to relate to faster infant reaction time [46]. Symbolic play with objects emerges during development in a hierarchical sequence of stages, with increasing complexity at each stage [47]. We assessed complexity of play using the procedure developed by Belsky and colleagues [48] and adapted by Jacobson and colleagues [49]. First, 10 min of free play with a standard set of toys is observed. Suggestion and modeling are subsequently utilized to elicit progressively higher levels of play complexity than those spontaneously demonstrated by the infant. Complexity of elicited play is scored as the highest of 14 levels successfully imitated by the infant. Infant temperament was assessed by maternal report at 12 months postpartum using the Emotionality, Activity and Sociability (EAS) Temperament Survey, a 20-item questionnaire, which assesses emotionality, activity, sociability, and shyness [50]. Each of these dimensions of temperament is measured by five items (e.g., tends to be shy, cries easily, likes to be with people), each of which is rated on a 5-point scale.

### 2.7. Statistical Analyses

All statistical analyses were conducted using SPSS (v.24; IBM, Armonk, NY, USA) and Stata (v.16.1; Statcorp, College Station, TX, USA). All variables were examined for normality of distribution; oz AA/day and ferritin values were log-transformed due to skewness (>3.0). All missing data were treated with list-wise deletion. Analyses were two-sided (α = 0.05). Linear regression models were constructed to examine associations of PAE and iron indices to infant outcomes, first in univariate models and then in multivariable models adjusting for potential confounders. Potential confounders for growth outcomes were those associated with PAE-related growth restriction in previous studies [7]: maternal age, smoking (no. prenatal cigarettes/day), maternal education, and weeks gestation at delivery. Potential confounders for neurobehavioral and temperament outcomes were those related to a given outcome at *p* ≤ 0.10 in univariate regression models (see Table S1). Interaction effects between PAE and iron indices were examined by constructing linear regression models regressing the infant outcome on PAE, a given iron measure, a PAE by iron measure interaction term, and potential confounders. Given the low power of interaction terms in observational studies, interaction effects were inferred when the *p*-value for the interaction term was ≤0.10 [51]. For any model with evidence of a PAE by iron measure interaction effect, two sets of stratified results were then examined: (1) the relation of the iron measure to the given outcome stratified by alcohol exposure (controls vs. heavy exposure); (2) the relation of PAE to the outcome stratified by the given iron measure (tertiles for continuous measures, yes vs. no for categorical variables (e.g., IDA)). To examine the hypothesis that relations of PAE to infant growth and neurobehavioral outcomes are mediated by alterations in iron indices, mediation analysis was conducted based on marginal structural models and the product method (paramed package, Stata) for variable triads where PAE (*exposure*) and a previously identified [12] PAE-related iron measure (*potential mediator*) were both related to an infant *outcome* [52,53]. This method allows for estimation of natural direct effects (exposure→outcome, independent of the potential mediator), natural indirect effects (exposure→outcome, through the potential mediator), and total effects with exposure-mediator interactions, with control for covariates from the relevant regression models described above. PAE-mediator interaction was modeled with drinking frequency increasing from 0 to 3 days/week to mimic the weekend drinking pattern seen in this cohort.

## 3. Results

### 3.1. Sample Characteristics

Women were in their 3rd decade on average, and heavy drinking mothers were 2.4 years older than controls (Table 1). Both heavy drinking and control mothers were poorly educated with heavy drinking women averaging 0.7 fewer years of school than controls. Heavy drinking mothers averaged 4.3 oz (129.0 mL) absolute alcohol (~7.2 standard drinks) per occasion on 1.4 days per week across pregnancy. Among drinking mothers, binge drinking was very common, with 93.1% averaging ≥2 oz (60 mL) AA/occasion. Controls abstained from drinking during pregnancy except for 8 women. These 8 all reported light drinking with no binges; 4 women reported 1–3 drinking occasions across pregnancy; 2 women reported monthly drinking occasions; 1 reported drinking once a week; and 1 drank 2.1 oz (63 mL) absolute alcohol (~3.5 drinks) on one occasion. Although cigarette smoking was more common among heavy drinking women than controls, number of cigarettes/day was similarly low in both groups (86.7% < 0.5 pack/day; 1.9% ≥ 1.0 pack/day). Co-exposures were common; 0.7% of heavy drinking women reported marijuana use vs. 11% of controls; 13.8% reported methamphetamine use vs. 15.5% of control women; methaqualone, cocaine, and opiate use were not reported by any participant. PAE was not associated with weeks gestation at delivery. Most women (86.7%) delivered at term. Infants born to heavy drinking mothers were more likely to be small for gestational age and were smaller than control infants across almost every anthropometric measure in the neonatal period and at 5 years.

**Table 1:** Sample characteristics.

	Controls (*n* = 71)		Heavy Drinking Mothers (*n* = 87)		
	*n*	*M* (SD) or *n* (%)	*n*	*M* (SD) or *n* (%)	*p* ^a^
Maternal characteristics					
Maternal age at conception (years)	71	25.5 (4.9)	87	27.9 (6.1)	0.003
Gravidity (no. live births (%))	71	1.5 (1.3)	87	1.8 (1.5)	0.090
Education (years school completed)	70	9.9 (1.7)	87	9.3 (1.6)	0.007
Marital status, no. married (%)	71	30 (42.3)	87	25 (28.7)	0.076
Weeks gestation at maternal visit 1	71	26.4 (5.2)	87	25.0 (5.6)	0.056
Weeks gestation at maternal visit 2	44	34.6 (3.8)	55	33.5 (4.1)	0.083
Iron intake from diet only (mg/day) ^b^	71	12.4 (3.7)	87	11.9 (2.9)	0.188
*n* (%) with inadequate intake ^c^	71	67 (94.4)	87	86 (98.9)	0.109
Iron/folic acid supplementation, *n* (%)	70	58 (82.9)	85	72 (84.7)	0.755
Took supplement on “most days”	58	56 (96.6)	72	70 (97.2)	0.826
Alcohol and drug use					
Alcohol consumption:	71		87		
oz AA/day		0.0 (0.0)		0.9 (1.2)	<0.001
oz AA/drinking day		0.1 (0.4)		4.3 (2.4)	<0.001
Drinking days/week		0.0 (0.1)		1.4 (1.1)	<0.001
*n* (%) reporting cigarette smoking	71	49 (69.0)	87	71 (81.6)	0.065
Cigarettes/day among smokers	49	5.8 (3.8)	71	6.7 (4.4)	0.121
*n* (%) reporting marijuana use	71	8 (11.3)	87	18 (20.7)	0.026
Marijuana days/month among users	8	4.0 (4.7)	18	9.7 (9.0)	0.047
*n* (%) reporting methamphetamine use	71	11 (15.5)	87	12 (13.8)	0.763
Methamphetamine days/month among users	11	10.2 (8.9)	12	4.3 (5.4)	0.033
Infant characteristics					
Weeks gestation at delivery	71	39.1 (1.8)	87	38.8 (2.1)	0.223
Preterm delivery (<37 weeks), *n* (%)		9 (12.7)		12 (13.8)	0.837
Infant sex, no. female (%)	71	33 (46.5)	87	45 (51.7)	0.512
Infant birthweight (g)	70	3078.8 (534.5)	87	2885.4 (609.5)	0.019
No. small for gestational age (%) ^d^	70	13 (18.6)	84	30 (35.7)	0.018
2-week *z*-scores ^e^					
Length	61	−1.3 (1.3)	69	−1.7 (1.4)	0.083
Weight	61	−0.8 (1.2)	69	−1.3 (1.4)	0.029
Head circumference	61	−0.4 (1.2)	69	−0.9 (1.5)	0.016
5-year *z*-scores ^e^					
Height	67	−0.5 (0.9)	80	−1.0 (0.9)	0.005
Weight	67	−0.2 (1.0)	80	−0.9 (0.8)	<0.001
5-year head circumference (cm)	67	50.3 (1.6)	80	48.7 (3.4)	<0.001

AA = absolute alcohol; 1 oz AA = 30 mL AA = 1.67 standard drinks. ^a^ From independent samples *t*-tests for continuous outcomes, chi-square for binary outcomes. ^b^ From three multipass 24-h recall dietary interviews. ^c^ Defined as dietary iron intake <23 mg/day if age <19 years; <22 mg/day if age 19 years and older [54]. ^d^ Defined as birthweight <10th percentile for gestational age [55]. ^e^ Age- and sex-specific *z*-scores from World Health Organization norms [43].

### 3.2. Relation of Prenatal Alcohol Exposure and Maternal and Infant Iron Indices to Neonatal and Postnatal Anthropometric Measures

As we have previously reported [12], PAE was related to smaller length- and weight-for-age *z*-scores at age 2 weeks (Table 2A). Each additional day/week of prenatal drinking was associated with a reduction of 0.28 SD for length and 0.21 SD for weight. PAE was associated with smaller head circumference-for-age *z*-scores at age 2 weeks in univariate analyses, but this relation was no longer statistically significant after adjusting for potential confounders. At age 5 years (Table 2B), PAE was related to smaller length and weight *z*-scores as well as smaller head circumference (cm). For length, the magnitude of effect was slightly larger at 2 weeks when compared to age 5 years (B = −0.28 and −0.19, respectively) and remarkably similar at the two ages for weight (B = −0.21 and −0.22, respectively). Each day/week of prenatal drinking was associated with a 5-year head circumference reduction of 0.73 cm.

Higher maternal prenatal ferritin (an indicator of iron in storage) and lower hemoglobin-to-log(ferritin) ratio [a measure of iron available for erythropoiesis (hemoglobin) vs. iron in storage (ferritin)] were both associated with smaller weight, length, and head circumference *z*-scores at age 2 weeks (Table 2A). A trend (*p* < 0.10) was seen for smaller length among infants whose mothers had anemia of inflammation during pregnancy. Higher infant hemoglobin at age 2 weeks was associated with larger weight, length, and head circumference *z*-scores in multivariable models. Higher maternal prenatal ferritin and lower hemoglobin-to-log(ferritin) ratio were associated with smaller height and weight *z*-scores at age 5 years (Table 2B). Neonatal hemoglobin-to-log(ferritin) ratio was positively associated with 5-year height *z*-scores. No other infant iron indices were related to infant anthropometric measures.

### 3.3. Relation of Prenatal Alcohol Exposure and Maternal and Infant Iron Indices to Infant Neurobehavior and Temperament

PAE was related to poorer visual recognition memory at age 6.5 months in univariate analyses and multivariable models adjusting for potential confounders (Table 3A); this association was not seen at age 12 months. At age 12 months PAE was associated with longer processing time, i.e., slower processing speed, and lower complexity of symbolic play. PAE was also associated with greater emotionality and shyness on the EAS temperament scales (Table 3B).

Higher maternal prenatal hemoglobin was associated with shorter 6.5-month processing time, i.e., faster processing speed. Higher neonatal ferritin was associated with poorer visual recognition memory at 12 months (Table 3A). Lower 6.5-month ferritin and the presence of ID at 6.5 months were both associated with longer 12-month processing time. Univariate associations between presence of maternal anemia of inflammation, neonatal hemoglobin, and 6.5-month ferritin ID, and IDA with symbolic play performance were no longer seen after adjustment for potential confounders. Higher maternal prenatal hemoglobin was associated with decreased sociability and increased shyness (Table 3B). A trend (*p* < 0.10) was seen for higher neonatal ferritin relating to increased emotionality. Higher 6.5-month ferritin was associated with increased activity. ID at age 6.5 months was associated with increased emotionality, decreased activity, and increased shyness; IDA was also associated with decreased activity.

### 3.4. Interaction Effects between Prenatal Alcohol Exposure and Maternal and Infant Iron Indices on Growth, Neurobehavior, and Temperament

Results from regression analyses examining potential interaction effects for PAE by maternal and infant iron indices on growth and neurobehavioral outcomes are presented in Supplementary Table S2. Table 4 displays stratified models for iron indices with evidence of interaction effects with PAE. Since interaction effects are 2-way, two sets of stratified results are presented: (1) the relation of the iron measure to the given outcome stratified by alcohol exposure (controls vs. heavy exposure); (2) the relation of PAE to the outcome stratified by the given iron measure (tertiles for continuous measures, yes vs. no for categorical outcomes (e.g., IDA). Higher maternal and neonatal hemoglobin-to-log(ferritin) ratios were both associated with larger neonatal length *z*-scores among exposed infants but not controls. Higher neonatal hemoglobin and hemoglobin-to-log(ferritin) ratio were both associated with higher neonatal weight *z*-scores among exposed infants but not controls. A trend for lower weight *z*-scores among exposed infants vs. controls was seen among infants in the lower third for neonatal hemoglobin that was not seen in the other groups. A trend for higher weight *z*-scores among exposed infants vs. controls was seen among infants in the upper third for neonatal hemoglobin-to-log(ferritin) ratio that was not seen in the other groups. Among exposed infants but not controls, higher maternal ferritin and lower maternal hemoglobin-to-log(ferritin) ratio were associated with smaller neonatal head circumference *z*-scores. PAE was related to smaller neonatal head circumference *z*-scores among infants whose mothers were in the upper third for maternal ferritin but not in the other groups. At age 5 years, the relation of PAE to lower height *z*-scores was seen only among infants whose mothers were in the lowest third for maternal hemoglobin, and paradoxically, higher maternal hemoglobin was associated with lower height *z*-scores among controls. Higher neonatal hemoglobin-to-log(ferritin) ratio was related to larger 5-year height *z*-scores among exposed but not control infants; PAE was related to smaller height *z*-scores among children in the lowest third for neonatal hemoglobin-to-log(ferritin) ratio but not among the other infants.

A trend (*p* < 0.10) association between higher maternal ferritin and better 6.5-month visual recognition memory was seen among control but not exposed infants (Table 4). A negative association between PAE and 6.5-month visual recognition memory was only seen among infants whose mothers were in the highest third for ferritin. A trend (*p* < 0.10) association between higher maternal hemoglobin and better 12-month visual recognition memory was seen among exposed infants but not controls, and the association of PAE to poorer 12-month visual recognition memory was only seen among infants whose mothers were in the lower third for hemoglobin. Higher maternal hemoglobin was associated with shorter processing time (i.e., faster processing speed) at 6.5 months among exposed but not control infants, and a trend (*p* < 0.10) association between PAE and longer processing time at this age was only seen among infants whose mothers were in the lower third for hemoglobin. An association between ID at 6.5 months and longer processing time (i.e., slower processing speed) at 6.5 months was seen among exposed but not control infants.

### 3.5. Alterations in Maternal and Infant Iron Homeostasis as Potential Mediators in Fetal Alcohol-Related Growth and Neurobehavioral Deficits

Table 5 presents the results of causal inference analyses using marginal structural models and the product method to examine the degree to which PAE-related alterations in maternal and infant iron homeostasis statistically mediate the relations between PAE and growth and neurobehavioral outcomes. These analyses were conducted for variable triads that consisted of PAE, an iron homeostasis outcome we have previously shown to be related to PAE [12], and a growth or neurobehavioral outcome related to both PAE and the given iron homeostasis outcome. When examining the PAE-related elevation in maternal ferritin as a potential mediator of the relation of PAE to neonatal length *z*-scores, the indirect effect of PAE through maternal ferritin comprised 33.3% of the total effect of PAE on neonatal length. Similarly, the indirect effect of PAE through maternal hemoglobin-to-log(ferritin) ratio comprised 82.3% of the total effect of PAE on neonatal length *z*-scores. When examining neonatal weight *z*-scores, a trend (*p <* 0.10) for an indirect effect of PAE through maternal ferritin comprised 32.8% of the total effect of PAE on weight. A trend (*p* < 0.10) for an indirect effect of PAE through maternal ferritin comprised 43.5% of the total effects of PAE on head circumference *z*-scores. At age 5 years, the indirect effect of PAE through maternal ferritin comprised 27.1% of the total effects of PAE on height *z*-scores. The indirect effect of PAE through maternal ferritin comprised 29.4% of the total effects of PAE on weight *z*-scores, and the indirect effect of PAE through maternal hemoglobin-to-log(ferritin) ratio comprised 22.6% of total effects on weight *z*-scores. Evidence of statistical mediation was not seen for neurobehavioral outcomes.

## 4. Discussion

In this prospective longitudinal birth cohort with serial measures of maternal, neonatal, and postnatal iron measures as well as serial child growth and neurobehavioral outcomes, PAE and maternal and infant iron measures had separate and overlapping associations with growth and neurobehavioral outcomes. Among the overlapping associations, PAE-related elevations in maternal ferritin and hemoglobin:log(ferritin) appeared to statistically mediate 22.6–82.3% of the effects of PAE on neonatal and postnatal anthropometric measures. Furthermore, interaction effects were also evident between PAE and PAE-related maternal and infant iron measures on both growth and neurobehavioral deficits, indicating that PAE-related alterations in maternal and infant iron homeostasis appear to exacerbate the teratogenic effects of alcohol. These findings are also consistent with previous animal studies [17,33,57]. To our knowledge, this is the first examination of the effects of PAE and iron measures on both growth and neurobehavior in a human study.

As we and others have previously demonstrated [7,58,59], PAE is related to both fetal and postnatal growth restriction. We have previously reported that maternal alcohol consumption is associated with elevated ferritin [12]. Given that PAE was also related to a lower hemoglobin-to-log(ferritin) ratio, we posited that PAE-related elevations in ferritin were not an indication of better overall iron status but rather represented iron being sequestered into storage at the expense of other physiologic processes, such as erythropoiesis and placental iron transport, as is seen in an inflammatory state. Consistent with this hypothesis, we found that higher maternal ferritin and lower maternal hemoglobin-to-log(ferritin) ratio were related to smaller neonatal length, weight, and head circumference and smaller 5-year height and weight. We have previously reported that PAE was associated with lower hemoglobin in this cohort, and in the analyses presented here, lower hemoglobin was related to smaller neonatal length, weight, and head circumference. Lower neonatal hemoglobin-to-log(ferritin) ratio, which we have shown to be associated with PAE, was related to lower weight at 5 years. In causal inference analyses, PAE-related elevations in maternal ferritin and reductions in maternal hemoglobin-to-log(ferritin) ratio appeared to partially statistically mediate effects of PAE on pre- and postnatal growth, indicating that these alterations in maternal iron homeostasis may play possible mechanistic roles in PAE-related pre- and postnatal growth restriction. These statistical mediation findings are consistent with previous animal and human studies demonstrating that reduced fetal iron stores may result in growth restriction [31,32]. Furthermore, interaction effects between PAE and maternal ferritin and maternal and neonatal hemoglobin-to-log(ferritin) ratio were seen, indicating that the detrimental effects of these iron indices on fetal growth are only seen in the setting of PAE. Additionally, PAE-related elevations in maternal ferritin appeared to exacerbate PAE-related neonatal head circumference reductions, and PAE-related reductions in neonatal hemoglobin-to-log(ferritin) ratio appeared to exacerbate PAE-related 5-year weight reductions. We have previously shown that PAE-related postnatal height restriction is a biomarker for the severity of FASD neurocognitive deficits [10].

As we have demonstrated in birth cohorts in Detroit, USA [9], and Cape Town, South Africa [60], PAE was related to poorer visual recognition memory and slower processing speed. Recognition memory has been shown to be negatively affected by inadequate iron in early infancy [19,25,28]. In this cohort, higher neonatal ferritin, which we have shown to be associated with PAE [12], was related to poorer visual recognition memory at 12 months. Although higher ferritin may be an indicator of better overall iron status, in this cohort, we found that PAE was associated with a lower neonatal hemoglobin-to-log(ferritin) ratio that partially mediated later development of IDA at age 6.5 months. These findings suggest that, as seen in the mothers, the higher ferritin likely represented iron sequestered into storage, making it unavailable for other processes like erythropoiesis, and, possibly, brain development. PAE-related elevations in maternal ferritin appeared to exacerbate the detrimental effects of PAE on recognition memory in that the relation of PAE to visual recognition memory was only seen among infants whose mothers were in the highest tertile for ferritin. Maternal hemoglobin appeared to alter the timing of PAE-related neurobehavioral deficits. In the whole cohort, effects on visual recognition memory at 6.5 months were no longer evident at 12 months, suggesting a PAE-related developmental delay for this outcome. However, among infants whose mothers were in the lowest third for hemoglobin, PAE was also related to poorer visual recognition memory at 12 months, indicating that low maternal hemoglobin may prolong PAE-related visual recognition memory deficits.

Our finding that higher maternal hemoglobin was related to faster processing speed is consistent with previous studies demonstrating slower processing speed among infants born to anemic mothers [31,61]. Similarly, as seen in previous human studies of infant ID [29,62], ID at age 6.5 months was associated with slower processing speed. Interaction effects between PAE and alterations in maternal and infant iron homeostasis on processing speed were also seen. The relation of PAE to slower processing speed seen at 12 months in the whole cohort was also evident at 6.5 months among infants whose mothers were in the lowest tertile for maternal hemoglobin, indicating that lower maternal hemoglobin may make this deficit evident earlier in infancy. Additionally, the relation of IDA at 6.5 months to slower processing speed was only seen among infants with PAE.

As we have previously demonstrated [24,49], PAE was associated with lower complexity of symbolic play, a known precursor to language [63], and higher emotionality and shyness scores on the EAS temperament assessment. ID at 6.5 months was associated with higher emotionality, lower activity, and increased shyness. These findings confirm data from our prospective longitudinal cohort of infants in inner-city Detroit, where ID at 9 months was associated with increased shyness and decreased activity on the EAS and increased latency to engage with the examiner and decreased orientation-engagement, soothability, and positive affect on the Bayley Behavior Rating Scale [30]. Higher maternal hemoglobin was related to lower sociability and greater shyness; the significance of this finding is unclear and may be a chance finding, as infants with better iron status would be expected to be more socially engaged than infants with iron deficiency.

Our findings that higher maternal ferritin and lower maternal hemoglobin-to-log(ferritin) ratio partially statistically mediated PAE-related fetal and postnatal growth restriction suggest that correcting these alterations in maternal iron homeostasis may ameliorate some of the effects of PAE on growth; as this study was an observational study, true causation cannot be inferred, and randomized controlled intervention trials are needed to evaluate this hypothesis. In this cohort, we recently demonstrated that the effects of PAE on neonatal weight and length and postnatal head circumference were only apparent among children whose mothers were in the lowest and middle tertiles for dietary iron intake [38]. Our findings demonstrating that low maternal hemoglobin and PAE-related elevations in maternal ferritin may exacerbate PAE-related deficits in recognition memory and/or processing speed suggest that improving maternal iron status may also mitigate some of the detrimental effects of PAE on neurobehavior. In Smith and colleagues’ FASD rat model studies, PAE-related alterations in maternal and offspring iron status mediated PAE-related growth and neurobehavioral deficits, and prenatal iron supplementation resulted in amelioration of these deficits [15,17]. Notably, in our pilot feasibility clinical trial demonstrating beneficial effects of prenatal choline supplementation on growth and neurobehavior among heavy drinking pregnant women, no treatment effects were seen on iron or hematological measures [64], suggesting that choline alone is likely to be insufficient to ameliorate these teratogenic effects of PAE.

Although clear evidence of mediation in neurobehavioral and temperament outcomes was not seen in causal inference analyses in this study, we cannot conclude that alterations in maternal and/or infant iron homeostasis do not partially mediate PAE effects on these outcomes. While effects of PAE on neurobehavioral outcomes were seen in linear regression models (Table 3), the total effects of PAE (direct + indirect) on these outcomes in causal inference analyses were significant for only two outcomes. This study may, therefore, have been underpowered to utilize marginal structural models for these outcomes. Other less robust approaches for examining statistical mediation that are less sensitive to power, such as the Sobel or Clogg tests, could not be used here, as they are not valid when interaction effects between the predictor and mediator are present [65]. Furthermore, although results from causal inference analyses with marginal structural models in observational studies more closely approximate true causation than other approaches, true causation cannot be inferred from mediation findings in observational studies such as this one. Our relatively small sample size, measurement error surrounding exposure estimates, and potential model misspecification and residual confounding may have led to Type 2 (false negative) errors. To minimize the risk of Type 1 (false positive) errors, we have focused particularly on the overall pattern of findings seen when examining multiple iron and infant outcome measures.

## 5. Conclusions

In summary, our findings add to the growing evidence in animal and human studies that fetal Alcohol-Related alterations in iron homeostasis in the mother, fetus, and postnatal infant may play mechanistic roles in FASD. PAE-related elevations in maternal ferritin appeared to partially statistically mediate pre- and postnatal fetal alcohol growth restriction. Furthermore, interaction effects between PAE and PAE-related maternal and infant iron measures on both growth and neurobehavioral deficits indicated that PAE-related alterations in maternal and infant iron homeostasis may exacerbate the teratogenic effects of alcohol. These findings provide strong justification for conducting randomized clinical trials in humans to examine the potential for prenatal iron supplementation to mitigate the teratogenic effects of alcohol on growth and neurobehavior. Future experimental animal and/or cell culture models examining the molecular mechanisms by which PAE and iron homeostasis interact in FASD may also yield novel, important insights into how PAE disrupts fetal development.

## Figures and Tables

**Table 2:** Relation of prenatal alcohol exposure and maternal and infant iron indices to infant anthropometric measures.

A. 2-week Neonatal Measures (n = 61 Controls, 69 Heavily Exposed)						
	Length-for-Age *z*-score ^a^		Weight-for-Age *z*-score ^a^		Head Circumference-for-Age *z*-score ^a^	
	B_1_	B_2_	B_1_	B_2_	B_1_	B_2_
Maternal alcohol consumption						
Drinking days/week	−0.36 ** (−0.58, −0.14)	−0.28 ** (−0.47, −0.09)	−0.30 ** (−0.51, −0.08)	−0.21 * (−0.39, −0.03)	−2.84 *** (−4.36, −1.33)	−2.40 (−3.66, −1.14)
Maternal iron indices						
Dietary iron (mg/day)	0.05 (−0.03, 0.12)	0.04 (−0.02, 0.11)	0.05 (−0.02, 0.12)	0.05 (−0.01, 0.10)	0.05 (−0.03, 0.12)	0.04 (−0.03, 0.10)
Ferritin (logged ug/L values)	−0.69 *** (−1.02, −0.37)	−0.54 *** (−0.83, −0.24)	−0.56 *** (−0.87, −0.25)	−0.39 ** (−0.67, −0.11)	−0.63 *** (−0.95, −0.30)	−0.46 ** (−0.75, −0.17)
Hemoglobin (g/dL)	−0.15 (−0.38, 0.08)	−0.03 (−0.23, 0.17)	−0.14 (−0.36, 0.08)	−0.01 (−0.20, 0.18)	−0.13 (−0.36, 0.11)	0.01 (−0.18, 0.21)
Hemoglobin:log(ferritin)	0.45 *** (0.19, 0.71)	0.34 ** (0.11, 0.57)	0.37 ** (0.12, 0.62)	0.26 * (0.05, 0.48)	0.46 *** (0.20, 0.72)	0.35 ** (0.12, 0.57)
Anemia of inflammation ^b^	−0.68 (−1.68, 0.32)	−0.72 ^†^ (−1.56, 0.11)	−0.38 (−1.35, 0.59)	−0.42 (−1.22, 0.38)	0.30 (−0.40, 1.01)	−0.51 (−1.32, 0.30)
Neonatal iron indices						
Ferritin (logged ug/L values)	0.03 (−0.35, 0.42)	−0.02 (−0.32, 0.29)	0.04 (−0.34, 0.41)	−0.04 (−0.33, 0.26)	0.17 (−0.23, 0.56)	0.13 (−0.17, 0.44)
Hemoglobin (g/dL)	0.10 ^†^ (−0.01, 0.21)	0.11 * (0.02, 0.20)	0.10 ^†^ (−0.01, 0.20)	0.10 * (0.02, 0.19)	0.08 (−0.03, 0.19)	0.09 * (0.00, 0.17)
Hemoglobin:log(ferritin)	0.31 (−0.17, 0.79)	0.33 ^†^ (−0.04, 0.70)	0.32 (−0.15, 0.79)	0.34 (−0.02, 0.71)	0.14 (−0.34, 0.62)	0.17 (−0.17, 0.51)
B. 5-year Postnatal Measures (*n* = 67 Controls, 80 Heavily Exposed)						
	Height-for-Age *z*-score ^a^		Weight-for-Age *z*-score ^a^		Head Circumference (cm)	
	B_1_	B_2_	B_1_	B_2_	B_1_	B_2_
Maternal alcohol consumption						
Drinking days/week	−0.24 *** (−0.38, −0.10)	−0.19 ** (−0.33, −0.05)	−0.29 *** (−0.43, −0.15)	−0.22 ** (−0.36, −0.08)	−0.70 *** (−1.13, −0.28)	−0.73 *** (−1.17, −0.29)
Maternal iron indices						
Dietary iron (mg/day)	−0.02 (−0.06, 0.03)	−0.02 (−0.07, 0.02)	0.01 (−0.04, 0.05)	0.00 (−0.05, 0.04)	−0.06 (−0.20, 0.08)	−0.09 (−0.24, 0.05)
Ferritin (logged ug/L values)	−0.37 *** (−0.58, −0.17)	−0.33 ** (−0.55, −0.11)	−0.45 *** (−0.66, −0.25)	−0.40 *** (−0.62, −0.19)	−0.43 (−1.09, 0.22)	−0.29 (−1.03, 0.44)
Hemoglobin (g/dL)	−0.14 * (−0.29, 0.00)	−0.10 (−0.25, 0.04)	−0.15 * (−0.30, 0.00)	−0.09 (−0.23, 0.05)	0.14 (−0.31, 0.58)	−0.29 (−1.03, 0.44)
Hemoglobin:log(ferritin)	0.23 ** (0.07, 0.39)	0.19 * (0.02, 0.35)	0.30 *** (0.14, 0.46)	0.25 ** (0.09, 0.41)	0.36 (−0.14, 0.85)	0.26 (−0.28, 0.79)
Anemia of inflammation ^b^	−0.42 (−1.01, 0.17)	−0.21 (−0.80, 0.38)	−0.57 ^†^ (−1.18, 0.04)	−0.31 (−0.89, 0.28)	−0.92 (−2.80, 0.96)	−0.89 (−2.86, 1.07)
Neonatal iron indices						
Ferritin (logged ug/L values)	−0.18 (−0.43, 0.07)	−0.23 ^†^ (−0.47, 0.01)	−0.03 (−0.29, 0.24)	−0.07 (−0.31, 0.18)	0.21 (−0.62, 1.05)	0.15 (−0.70, 1.00)
Hemoglobin (g/dL)	0.03 (−0.04, 0.10)	0.03 (−0.04, 0.10)	0.03 (−0.05, 0.10)	0.03 (−0.04, 0.11)	0.08 (−0.15, 0.32)	0.09 (−0.16, 0.35)
Hemoglobin:log(ferritin)	0.30 ^†^ (−0.01, 0.61)	0.30 * (0.00, 0.60)	0.13 (−0.20, 0.46)	0.14 (−0.17, 0.46)	0.23 (−0.83, 1.28)	0.26 (−0.82, 1.34)
6.5-month infant iron indices	0.19 (−0.01, 0.39)	0.08 (−0.12, 0.28)	0.14 (−0.07, 0.35)	−0.01 (−0.21, 0.19)	0.20 (−0.44, 0.84)	0.13 (−0.55, 0.81)
Ferritin (logged ug/L values)	−0.05 ^†^ (−0.20, 0.09)	−0.05 (−0.19, 0.09)	−0.06 (−0.21, 0.10)	−0.06 (−0.20, 0.08)	0.13 (−0.32, 0.58)	0.17 (−0.29, 0.63)
Hemoglobin (g/dL)	−0.28 (−0.63, 0.07)	−0.13 (−0.48, 0.22)	−0.12 (−0.49, 0.24)	0.08 (−0.26, 0.42)	−0.01 (−1.10, 1.09)	0.13 (−1.01, 1.26)
Iron deficiency ^b^	−0.08 (−0.55, 0.40)	−0.03 (−0.50, 0.45)	0.00 (−0.52, 0.52)	0.08 (−0.26, 0.42)	0.01 (−0.79, 0.80)	0.08 (−0.69, 0.85)
Iron deficiency anemia ^b^	0.19 (−0.25, 0.63)	0.05 (−0.38, 0.48)	0.10 (−0.36, 0.56)	−0.06 (−0.50, 0.38)	0.18 (−0.63, 0.98)	−0.08 (−0.87, 0.72)

B_1_ = regression coefficients (95% CI) from univariate linear regression models, B_2_ = regression coefficients (95% CI) from multivariable linear regression models adjusting for potential confounders (maternal age, prenatal cigarettes/day, maternal education, and weeks gestation at delivery for all outcomes, with the addition of age at time of measurement for 5-year head circumference), ^a^ Age/sex-specific *z*-scores from World Health Organization norms [43]. ^b^ Yes = 1; no = 0. ^†^ *p* ≤ 0.10; * *p* ≤ 0.05; ** *p* ≤ 0.01; *** *p* ≤ 0.001.

**Table 3:** Relation of prenatal alcohol exposure and maternal and infant iron indices to infant neurobehavior and temperament.

A. Neurobehavior (*n* = 71 Controls, 83 Heavily Exposed at 6.5 months; 70 Controls, 81 Heavily Exposed at 12 months)										
	Visual Recognition Memory ^a^				Information Processing Time (ms) ^a^				Symbolic Play (12 months) ^b^	
	6.5 months		12 months		6.5 months		12 months			
	B_1_	B_2_	B_1_	B_2_	B_1_	B_2_	B_1_	B_2_	B_1_	B_2_
Maternal alcohol consumption										
Drinking days/week	−1.39 ** (−2.43, 0.34)	−1.22 * (−2.29, −0.14)	−0.50 (−1.60, 0.60)	−0.53 (−1.68, 0.61)	0.01 (−0.07, 0.08)	0.01 (−0.07, 0.08)	0.08 * (0.01, 0.15)	0.08 * (0.01, 0.15)	−0.61 * (−1.12, −0.06)	−0.62 * (−1.2, −0.10)
Maternal iron indices										
Dietary iron (mg/day)	−0.17 (−0.52, 0.17)	−0.23 (−0.57, 0.12)	−0.11 (−0.48, 0.26)	−0.11 (−0.49, 0.27)	0.00 (−0.02, 0.03)	0.00 (−0.02, 0.03)	0.02 ^†^ (0.00, 0.05)	0.02 ^†^ (0.00, 0.05)	−0.12 (−0.32, 0.05)	−0.09 (−0.26, 0.09)
Ferritin (logged ug/L values)	−1.10 (−2.54, 0.34)	−0.66 (−2.20, 0.89)	0.00 (−1.61, 1.61)	−0.05 (−1.79, 1.68)	−0.05 (−0.15, 0.05)	−0.05 (−0.15, 0.05)	0.08 (−0.02, 0.19)	0.09 (−0.01, 0.20)	−0.15 (−0.96, 0.67)	0.00 (−0.81, 0.80)
Hemoglobin (g/dL)	−0.36 (−1.44, 0.73)	−0.16 (−1.27, 0.95)	0.27 (−0.84, 1.38)	0.22 (−0.95, 1.40)	−0.07 * (−0.15, 0.00)	−0.08 * (−0.16, 0.01)	0.02 (−0.05, 0.09)	0.02 (−0.05, 0.10)	0.22 (−0.35, 0.78)	0.22 (−0.32, 0.75)
Hemoglobin:log(ferritin)	−0.50 (−0.62, 1.62)	−0.12 (−1.06, 1.30)	0.08 (−1.13, 1.28)	0.09 (−1.18, 1.36)	−0.01 (−0.09, 0.07)	−0.02 (−0.10, 0.06)	−0.06 (−0.14, 0.02)	−0.06 (−0.14, 0.02)	−0.20 (−0.41, 0.82)	0.04 (−0.56, 0.64)
Anemia of inflammation ^c^	−1.28 (−5.77, 3.21)	−0.23 (−4.82, 4.37)	−1.40 (−6.24, 3.43)	−1.46 (−6.47, 3.55)	0.02 (−0.30. 0.34)	0.05 (−0.27, 0.36)	0.24 (−0.07, 0.56)	0.26 (−0.05, 0.57)	−2.32 * (−4.51, −0.11)	−1.63 (−3.80, 0.54)
Neonatal iron indices										
Ferritin (logged ug/L values)	−0.35 (−2.23, 1.54)	−0.27 (−2.13, 1.60)	−2.13 * (−4.16, −0.10)	−2.27 * (−4.35, −0.18)	0.01 (−0.10, 0.11)	0.01 (−0.10, 0.11)	−0.05 (−0.18, 0.08)	−0.01 (−0.18, 0.08)	0.36 (−0.64, 1.36)	0.10 (−0.87, 1.06)
Hemoglobin (g/dL)	0.25 (−0.27, 0.76)	0.26 (−0.26, 0.78)	−0.41 (−0.96, 0.14)	−0.45 (−1.02, 0.13)	0.00 (−0.03, 0.03)	0.00 (−0.03, 0.03)	−0.01 (−0.05, 0.02)	−0.04 (−0.17, 0.08)	0.36 ** (0.09, 0.63)	0.25 (−0.02, 0.52)
Hemoglobin:log(ferritin)	1.60 (−0.83, 4.04)	1.39 (−0.99, 3.76)	0.27 (−2.32, 2.86)	0.30 (−2.35, 2.95)	−0.03 (−0.16, 0.10)	−0.02 (−0.15, 0.11)	0.01 (−0.15, 0.17)	0.01 (−0.15, 0.17)	1.10 ^†^ (−0.18, 2.37)	0.95 ^†^ (−0.25, 2.15)
6.5-month infant iron indices										
Ferritin (logged ug/g values)	−0.25 (−1.74, 1.24)	−0.56 (−2.06, 0.94)	−0.71 (−2.23, 0.82)	−0.82 (−2.41, 0.77)	0.09 (−0.02, 0.19)	0.09 (−0.02, 0.19)	−0.11 * (−0.21, −0.01)	−0.11 * (−0.20, −0.01)	1.00 ** (0.25, 1.75)	0.39 (−0.40, 1.18)
Hemoglobin (g/dL)	−0.51 (−1.54, 0.52)	−0.52 (−1.55, 0.52)	0.03 (−1.07, 1.13)	−0.05 (−1.19, 1.09)	−0.05 (−0.13, 0.02)	−0.05 (−0.12, 0.03)	0.01 (−0.06, 0.08)	0.02 (−0.05, 0.09)	0.19 (−0.37, 0.74)	0.08 (−0.45, 0.61)
Iron deficiency ^c^	−0.29 (−2.72, 2.13)	0.22 (−2.21, 2.65)	1.00 (−1.61, 3.61)	1.13 (−1.55, 3.80)	−0.03 (−0.20, 0.14)	−0.03 (−0.20, 0.14)	0.21 ** (0.04, 0.37)	0.21 ** (0.04, 0.37)	−1.42 * (−2.73, −0.12)	−0.69 (−1.99, 0.61)
Iron deficiency anemia ^c^	−0.82 (−3.83, 2.18)	−0.53 (−3.51, 2.45)	0.11 (−3.13, 3.35)	0.23 (−3.05, 3.52)	0.19 ^†^ (−0.02, 0.4)	0.18 ^†^ (−0.03, 0.39)	0.13 (−0.08, 0.33)	0.13 (−0.08, 0.33)	−1.74 * (−3.73, −0.10)	−1.18 (−2.77, 0.40)
B. Temperament (*n* = 70 Controls, 81 Heavily Exposed)										
	EAS Temperament Scale ^d^ (12 months)									
	Emotionality			Activity		Sociability			Shyness	
	B_1_		B_2_	B_1_	B_2_	B_1_		B_2_	B_1_	B_2_
Maternal alcohol consumption										
Drinking days/week	0.10 ** (0.04, 0.17)		0.09 ** (0.03, 0.15)	−0.02 (−0.10, 0.05)	−0.02 (−0.09, 0.05)	−0.05 (−0.13, 0.02)		−0.04 (−0.11, 0.04)	0.09 * (0.01, 0.17)	0.08 * (0.00, 0.16)
Maternal iron indices										
Dietary iron (mg/day)	0.01 (−0.01, 0.03)		0.01 (−0.01, 0.04)	0.00 (−0.02, 0.02)	−0.00 (−0.02, 0.02)	−0.01 (−0.04, 0.01)		−0.02 (−0.04, 0.01)	0.00 (−0.02, 0.03)	0.00 (−0.02, 0.03)
Ferritin (logged ug/L values)	0.09 ^†^ (−0.01, 0.18)		0.07 (−0.03, 0.16)	0.02 (0.08, −0.12)	0.0 (−.10, 0.10)	−0.09 ^†^ (−0.19, 0.02)		−0.05 (−0.15, 0.06)	0.04 (−0.08, 0.15)	0.03 (−0.09, 0.14)
Hemoglobin (g/dL)	0.04 (−0.03, 0.10)		0.03 (−0.03, 0.10)	0.03 (−0.04, 0.11)	0.02 (−0.05, 0.10)	−0.10 ** (−0.17, −0.02)		−0.09 * (−0.16, −0.01)	0.08 * (0.00, 0.16)	0.08 * (0.00, 0.16)
Hemoglobin:log(ferritin)	−0.04 (−0.11, 0.04)		−0.02 (−0.09, 0.05)	0.00 (−0.08, 0.08)	0.02 (−0.06, 0.09)	0.04 (−0.04, 0.12)		0.01 (−0.07, 0.09)	0.00 (−0.08, 0.09)	0.01 (−0.07, 0.10)
Anemia of inflammation ^c^	0.29 * (0.00, 0.57)		0.27 ^†^ (−0.02, 0.55)	−0.03 (−0.34, 0.29)	−0.03 (−0.33, 0.28)	−0.03 (−0.35, 0.30)		0.01 (−0.31, 0.33)	0.04 (−0.32, 0.40)	0.01 (−0.35, 0.37)
Neonatal iron indices										
Ferritin (logged ug/L values)	0.11 * (0.00, 0.22)		0.11 ^†^ (−0.00, 0.22)	0.03 (−0.10, 0.16)	0.05 (−0.08, 0.18)	−0.06 (−0.20, 0.08)		−0.07 (−0.21, 0.07)	0.00 (−0.15, 0.14)	0.00 (−0.14, 0.15)
Hemoglobin (g/dL)	0.01 (−0.04, 0.03)		−0.01 (−0.04, 0.02)	0.01 (−0.03, 0.05)	0.01 (−0.02, 0.05)	0.00 (−0.04, 0.04)		0.00 (−0.04, 0.04)	−0.02 (−0.06, 0.02)	−0.02 (−0.06, 0.02)
Hemoglobin:log(ferritin)	−0.12 (−0.26, 0.03)		−0.12 (−0.26, 0.03)	−0.01 (−0.18, −0.16)	−0.03 (−0.19, 0.14)	0.05 (−0.12, 0.23)		0.07 (−0.11, 0.24)	0.66 (−0.89, 2.21)	0.56 (−0.98, 2.09)
6.5-month infant iron indices										
Ferritin (logged ug/L values)	−0.04 (−0.13, 0.05)		−0.04 (−0.13, 0.05)	0.09 ^†^ (−0.01, 0.19)	0.12 * (0.02, 0.22)	0.08 (−0.02, 0.18)		0.07 (−0.03, 0.17)	−0.06 (−0.17, 0.05)	−0.06 (−0.17, 0.05)
Hemoglobin (g/dL)	−0.04 (−0.10, 0.03)		−0.03 (−0.10, 0.03)	0.02 (−0.05, 0.09)	0.03 (−0.04, 0.10)	0.03 (−0.04, 0.10)		0.02 (−0.05, 0.10)	−0.01 (−0.09, 0.07)	−0.02 (−0.10, 0.06)
Iron deficiency ^c^	0.17 * (0.02, 0.33)		0.16 * (0.01, 0.31)	−0.19 * (−0.35, 0.02)	−0.22 ** (−0.39, −0.06)	−0.15 ^†^ (−0.32, 0.02)		−0.11 (−0.29, 0.06)	0.19 * (0.01, 0.38)	0.18 * (0.00, 0.37)
Iron deficiency anemia ^c^	0.11 (−0.09, 0.30)		0.09 (−0.10, 0.28)	−0.22 * (−0.42, −0.01)	−0.25 * (−0.45, −0.04)	−0.07 (−0.28, 0.14)		−0.04 (−0.25, 0.17)	−0.09 (−0.28, 0.10)	−0.07 (−0.26, 0.11)

B_1_ = regression coefficient (95% CI) from univariate linear regression models. B_2_ = regression coefficient (95% CI) from multivariable linear regression models including: maternal age, gravidity, and weeks gestation at delivery for visual recognition memory; prenatal marijuana use (days/month) for information processing time; maternal education, weeks gestation at delivery, infant sex, and age at time of assessment for symbolic play; gravidity for emotionality; socioeconomic status, prenatal marijuana use (days/month), infant sex for activity; gravidity and prenatal methamphetamine use (days/month) for sociability; prenatal cigarette smoking (cigarettes/day) and marijuana use (days/month) for shyness. ^a^ From the Fagan Test of Infant Intelligence [44,56]; missing for 2 controls and 8 heavily exposed infants at 6.5 months, 5 control and 2 heavily exposed infants at 12 months. ^b^ Highest level of imitated play complexity [48]; missing for 6 control and 3 heavily exposed infants. ^c^ Yes = 1; no = 0. ^d^ Emotionality, Activity, and Sociability temperament scale [50]. ^†^ *p* ≤ 0.10; * *p* ≤ 0.05; ** *p* ≤ 0.01.

**Table 4:** Stratified models demonstrating interaction effects between prenatal alcohol exposure and maternal and infant iron indices on growth and neurobehavior.

	Interaction Term *p* ^a^	Exposure Stratification Groups		Iron Measure Stratification Groups		
Neonatal length-for-age *z*-score ^b^ (age 2 weeks)		Controls	Heavy exposure	Lower third	Middle third	Upper third
		B_ind_	B_ind_	B_alc_	B_alc_	B_alc_
Maternal hb:log(ferritin) ^c^	0.105	0.09 (−0.25, 0.43)	0.45 ** (0.10, 0.80)	−0.33 (−1.52, 0.85)	−0.35 (−0.96, 0.26)	−0.04 (−0.50, 0.41)
Neonatal hb:log(ferritin) ^c^	0.021	−0.09 (−0.53, 0.36)	0.91 ** (0.23, 1.58)	−0.65 (−1.44, 0.15)	−0.17 (−0.94, 0.60)	0.45 (−0.34, 1.25)
Neonatal weight-for-age *z*-score ^a^ (age 2 weeks)		Controls	Heavy exposure	Lower third	Middle third	Upper third
		B_ind_	B_ind_	B_alc_	B_alc_	B_alc_
Neonatal hemoglobin (g/dL)	0.020	0.00 (−0.1, 0.1)	0.24 ** (0.08, 0.39)	−0.79 ^†^ (−1.63, 0.06)	−0.08 (−0.72, 0.58)	0.09 (−0.51, 0.69)
Neonatal hb:log(ferritin) ^c^	0.011	−0.11 (−0.53, 0.31)	0.93 ** (0.26, 1.60)	−0.59 (−1.54, 0.37)	−0.25 (−0.95, 0.45)	0.47 (−0.06, 1.00)
Neonatal head circumference-for-age *z*-score ^b^ (age 2 weeks)		Controls	Heavy exposure	Lower third	Middle third	Upper third
		B_ind_	B_ind_	B_alc_	B_alc_	B_alc_
Maternal ferritin (logged ug/L values) ^d^	0.028	0.14 (−0.31, 0.60)	−0.53 * (−0.96, −0.11)	−0.06 (−0.60, 0.49)	−0.37 (−1.14, 0.39)	−1.07 * (−1.99, −0.15)
Maternal hb:log(ferritin) ^c^	0.074	0.04 (−0.24, 0.32)	0.43 * (0.06, 0.79)	−0.57 (1.51, 0.37)	−0.28 (−1.01, 0.44)	−0.21 (−0.68, 0.26)
Postnatal height-for-age *z*-score ^b^ (age 5 years)		Controls	Heavy exposure	Lower third	Middle third	Upper third
		B_ind_	B_ind_	B_alc_	B_alc_	B_alc_
Maternal hemoglobin (g/dL)	0.081	−0.23 * (−0.44, −0.02)	0.01 (−0.18, 0.21)	−0.62 ** (−1.07, −0.17)	−0.28 (−0.89, 0.33)	−0.17 (−0.73, 0.40)
Neonatal hb:log(ferritin) ^c^	0.059	0.01 (−0.40, 0.42)	0.64 * (0.14, 1.1)	−0.71 * (−1.42, −0.01)	0.16 (−0.49 0.81)	−0.21 (−0.59, 0.17)
Visual recognition memory (6.5 months) ^e^		Controls	Heavy exposure	Lower third	Middle third	Upper third
		B_ind_	B_ind_	B_alc_	B_alc_	B_alc_
Maternal ferritin (logged ug/L values) ^d^	0.021	2.54 ^†^ (−0.35, 5.42)	−1.51 (−3.47, 0.45)	0.27 (−4.24, 4.78)	0.43 (−3.13, 3.98)	−7.15 *** (−11.35, −2.95)
Visual recognition memory (12 months) ^e^		Controls	Heavy exposure	Lower third	Middle third	Upper third
		B_ind_	B_ind_	B_alc_	B_alc_	B_alc_
Maternal hemoglobin (g/dL)	0.038	−0.74 (−2.09, 0.62)	1.58 ^†^ (−0.13, 3.29)	−4.60 * (−8.55, −0.67)	−0.21 (−3.78, 3.36)	−1.59 (−6.26, 3.09)
Information processing time (6.5 months) ^e^		Controls	Heavy exposure	Lower third	Middle third	Upper third
		B_ind_	B_ind_	B_alc_	B_alc_	B_alc_
Maternal hemoglobin (g/dL)	0.091	−0.03 (−0.14, 0.09)	−0.16 ** (−0.26, −0.06)	0.20 ^†^ (−0.03, 0.44)	−0.05 (−0.25, 0.16)	−0.06 (−0.40, 0.29)
		Controls	Heavy exposure	No iron deficiency anemia	Iron deficiency anemia	
		B_ind_	B_ind_	B_alc_	B_alc_	
Infant iron deficiency anemia at 6.5 months (Yes = 1, No = 0) ^d^	0.088	0.00 (−0.45, 0.45)	0.41 ** (0.16, 0.66)	−0.18 (−0.43, 0.07)	0.20 (−0.19, 0.59)	

B_ind_ = regression coefficient (95% CI) for the given iron measure regressed on the given outcome and potential confounders (maternal age, prenatal cigarettes/day, maternal education, and weeks gestation at delivery for anthropometry measures, with the addition of age at time of measurement for 5-year head circumference; maternal age, gravidity, and weeks gestation at delivery for visual recognition memory), stratified by heavy exposure vs. controls. B_alc_ = regression coefficient (95% CI) for prenatal alcohol exposure (heavy exposure = 1, control = 0) regressed on the given outcome and potential confounders (as listed above), stratified by the given iron measure. ^a^ *p*-value for the interaction term in a model regressing a given outcome on PAE, the given iron measure, a PAE x iron measure outcome, and potential confounders (please see Supplementary Table S2 for full results from these models). ^b^ Age/sex-specific *z*-scores from World Health Organization norms [43]; *n* = 61 controls, 69 heavily exposed at 2 weeks; 67 controls, 80 heavily exposed at 5 years. ^c^ Negatively related to prenatal alcohol exposure in previously published analyses [12]. ^d^ Positively related to prenatal alcohol exposure in previously published analyses [12]. ^e^ From the Fagan Test of Infant Intelligence [44,56]; *n* = 69 controls, 75 heavily exposed at 6.5 months; 65 controls, 79 heavily exposed at 12 months. ^†^ *p* ≤ 0.10; * *p* ≤ 0.05; ** *p* ≤ 0.01; *** *p* ≤ 0.001.

**Table 5:** Alterations in Maternal and Infant Iron Homeostasis as Potential Mediators in Fetal Alcohol-Related Growth and Neurobehavioral Deficits ^a^.

	Natural Direct Effect	Natural Indirect Effect	Total Effects	Proportion Mediation (%)
Neonatal length-for-age *z*-scores ^b^ (age 2 weeks)				
Maternal ferritin (logged ug/L values) ^c^	−0.52 ^†^ (−1.11, 0.08)	−0.26 * (−0.50, −0.03)	−0.78 ** (−1.35, −0.22)	33.3
Maternal hemoglobin:log(ferritin) ^d^	−0.11 (−0.98, 0.76)	−0.51 * (−1.04, 0.01)	−0.62 ^†^ (−1.26, 0.01)	82.3
Neonatal hemoglobin (g/dL)	−0.34 (−0.97, 0.29)	−0.09 (−0.23, 0.06)	−0.43 (−1.06, 0.20)	−−
Neonatal hemoglobin:log(ferritin) ^d^	0.25 (−0.59, 1.10)	−0.57 ^†^ (−1.20, 0.07)	−0.31 (−1.07, 0.45)	−−
Neonatal weight-for-age *z*-scores ^b^ (age 2 weeks)				
Maternal ferritin (logged ug/L values) ^c^	−0.38 (−0.96, 0.18)	−0.19 ^†^ (−0.40, 0.02)	−0.58 ** (−1.11, −0.04)	32.8
Maternal hemoglobin:log(ferritin) ^d^	−0.43 (−1.00, 0.14)	−0.15 (−0.35, 0.04)	−0.59 * (−1.12, 0.05)	−−
Neonatal hemoglobin (g/dL)	0.11 (−0.54, 0.75)	−0.31 (−0.75, 0.11)	−0.21 (−0.88, 0.46)	−−
Neonatal hemoglobin:log(ferritin) ^d^	0.69 ^†^ (−0.12, 1.50)	−0.68 ^†^ (−1.37, 0.00)	0.01 (−0.77, 0.79)	−−
Neonatal head circumference-for-age *z*-scores ^b^ (age 2 weeks)				
Maternal ferritin ^c^	−0.49 (−1.23, 0.26)	−0.37 ^†^ (−0.75, 0.01)	−0.85 ** (−1.44, −0.27)	43.5
Maternal hemoglobin:log(ferritin) ^d^	−0.55 (−1.38, 0.27)	−0.35 (−0.82, 0.12)	−0.90 ** (−1.49, −0.31)	−−
Postnatal length-for-age *z*-scores ^b^ (age 5 years)				
Maternal ferritin (logged ug/L values) ^c^	−0.43 * (−0.86, 0.00)	−0.16 * (−0.32, 0.00)	−0.59 ** (−1.01, −0.18)	27.1
Maternal hemoglobin (g/dL)	−0.62 ** (−1.04, −0.20)	0.00 (−0.12, 0.11)	−0.62 ** (−1.04, −0.20)	−−
Maternal hemoglobin:log(ferritin) ^d^	−0.48 * (−0.91, −0.05)	−0.11 (−0.25, 0.03)	−0.59 ** (−1.01, −0.17)	−−
Neonatal hemoglobin:log(ferritin) ^d^	−0.32 (−1.01, 0.38)	−0.39 (−0.87, 0.10)	−0.70 * (−1.32, −0.08)	−−
Postnatal weight-for-age *z*-scores ^b^ (age 5 years)				
Maternal ferritin ^c^	−0.48 * (−0.89, −0.01)	−0.20 * (−0.37, −0.04)	−0.68 *** (−1.09, −0.27)	29.4
Maternal hemoglobin:log(ferritin) ^d^	−0.52 ** (−0.94, −0.10)	−0.15 * (−0.30, −0.00)	−0.68 *** (−1.09, −0.27)	22.6
Visual recognition memory (6.5 months) ^e^				
Maternal ferritin (logged values) ^c^	−3.12 (−7.31, 1.06)	−0.39 (−2.37, 1.57)	−3.52 * (−6.85, −0.19)	−−
Visual recognition memory (12 months) ^e^				
Maternal hemoglobin (g/dL)	−1.41 (−4.95, 2.14)	−0.03 (−0.54, 0.48)	−1.44 (−5.01, 2.13)	−−
Neonatal ferritin				
Information processing time (6.5 months) ^e^				
Maternal hemoglobin (g/dL)	0.12 (−0.11, 0.21)	−0.04 (−0.16, 0.09)	0.09 (−0.16, 0.33)	−−
Infant iron deficiency anemia at 6.5 months ^c,g^	0.13 (−0.10, 0.35)	0.08 (−0.06, 0.21)	0.20 (−0.04, 0.45)	−−
Symbolic play (12 months) ^f^				
Infant iron deficiency anemia at 6.5 months ^c,g^	−0.97 (−2.82, 0.88)	−0.30 (−0.99, 0.38)	−1.27 (−2.14, 0.59)	−−
EASS emotionality (12 months) ^h^				
Infant iron deficiency ^c,g^	0.42 *** (0.20, 0.65)	0.03 (−0.02, 0.09)	0.46 *** (0.23, 0.68)	−−
EASS shyness (12 months) ^h^				
Infant iron deficiency ^c,g^	0.29 * (0.00, 0.59)	0.03 (−0.03, 0.08)	0.32 * (0.03, 0.61)	−−

^a^ Values are regression coefficients (95% confidence intervals) for prenatal alcohol exposure from marginal structural models and the product method for variable triads where PAE (*exposure*) and a previously identified [12] PAE-related iron measure (*potential mediator*) were both related to an infant *outcome* [52,53]. This method allows for estimation of natural direct effects (exposure→outcome, independent of the potential mediator), natural indirect effects (exposure→outcome, through the potential mediator), and total effects with exposure-mediator interactions, with control for potential confounders (covariates from the relevant regression models in Table 2 and Table 3). PAE-mediator interaction was modeled with drinking frequency increasing from 0 to 3 days/week to mimic the weekend binge-drinking pattern seen in this cohort. Proportion mediation (%) = natural indirect effect/total effects, calculated only where *p* ≤ 0.10 natural indirect effect or total effect. ^b^ Age/sex-specific *z*-scores from World Health Organization norms [43]; *n* = 61 controls, 69 heavily exposed at 2 week; 67 controls, 80 heavily exposed at 5 years. ^c^ Positively related to prenatal alcohol exposure in previously published analyses [12]. ^d^ Negatively related to prenatal alcohol exposure in previously published analyses [12]. ^e^ From the Fagan Test of Infant Intelligence [44,56]; *n* = 69 controls, 75 heavily exposed at 6.5 months; 65 controls, 79 heavily exposed at 12 months. ^f^ Highest level of imitated play complexity [48]; *n* = 64 controls, 78 heavily exposed. ^g^ Yes = 1; no = 0. ^h^ Emotionality, Activity, and Sociability temperament scale [50]; *n* = 70 controls, 81 heavily exposed. ^†^ *p* ≤ 0.10; * *p* ≤ 0.05; ** *p* ≤ 0.01; *** *p* ≤ 0.001.

## Data Availability

De-identified, individual participant data that underlie the results reported in this article and the study protocol, statistical analysis plan, and analytic code will be available for sharing to journal editors for any reason either before or after publication for checking and to researchers who provide a methodologically sound proposal, as determined by the authors of this article. Proposals from interested parties should be directed to Sandra W. Jacobson, PhD (sandra.jacobson@wayne.edu). Data will be stored in a data repository at Wayne State University and transmitted electronically in encrypted form to requestors. Data requestors will need to sign a data access agreement prior to access.

## Supplementary Materials

The following supporting information can be downloaded at: https://www.mdpi.com/article/10.3390/nu14204432/s1: Table S1. Relations of potential confounders to neurobehavioral outcomes; Table S2. Potential interaction effects between prenatal alcohol exposure and iron indices on infant growth, neurobehavior, and temperament.

## Abbreviations

AA = absolute alcohol; EAS = Emotionality, Activity, and Sociability; FAS = fetal alcohol syndrome; FASD = fetal alcohol spectrum disorders; ID = iron deficiency; IDA = iron deficiency anemia; PAE = prenatal alcohol exposure; PFAS = partial fetal alcohol syndrome.
