# Supplementary material for: Mediating and Moderating Effects of Iron Homeostasis Alterations on Fetal Alcohol-Related Growth and Neurobehavioral Deficits
Source: Nutrients. 2022 Oct 21;14(20):4432. doi: 10.3390/nu14204432 (PMC9607139; doi:10.3390/nu14204432)
Supplement: Supplementary file 1 [file nutrients-14-04432-s001.zip › nutrients-1920757-supplementary.pdf]

Supplementary Materials for:

Mediating and Moderating Effects of Iron Homeostasis Alterations on Fetal Alcohol–Related Growth and Neurobehavioral Deficits

R. Colin Carter, Neil C. Dodge, Christopher D. Molteno, Ernesta M. Meintjes, Joseph L. Jacobson and Sandra W. Jacobson

Table S1. Relation of potential confounders to neurobehavioral outcomes[a]

	2-wk weight[b]	2-wk length[b]	2-wk head circum-ference[b]	5-yr weight[b]	5-yr length[b]	5-yr head circum-ference	Visual recognition memory[c] (6.5 mo)	Visual recognition memory[c] (12 mo)	Mean look time[c] (6.5 mo)	Mean look time[c] (12 mo)	Symbolic play[d]	EAS[e] shy-ness	EAS[e] emotio-nality	EAS[e] socia-bility	EAS[e] act-ivity
Maternal age	-0.16†	-0.12	-0.17*	-0.14†	-0.11	-0.04	-0.15†	0.00	-0.11	0.06	-0.03	0.00	0.05	-0.10	0.00
Gravidity	-0.10	-0.05	-0.12	-0.13	-0.02	-0.04	-0.14†	-0.03	-0.01	0.01	-0.03	0.09	0.17*	-0.17*	0.03
Maternal education	0.26**	0.29***	0.17†	0.32***	0.23**	0.01	0.09	-0.03	0.03	-0.07	0.15†	0.08	0.02	0.02	0.00
Cigarettes/day	-0.23**	-.19*	-0.19*	-0.19*	-0.14†	0.03	0.02	-0.06	-0.12	0.08	0.07	0.15†	0.12	-0.08	0.02
Marijuana use	0.00	0.00	-0.02	-0.07	0.03	-0.09	-0.06	-0.13	0.18*	0.10	0.13	0.14†	0.11	-0.05	-0.17*
Methamphetamine use	-0.06	-0.10	-0.07	-0.02	-0.06	0.05	0.06	-0.03	-0.05	-0.02	0.10	-0.04	0.06	0.13†	0.01
Wk gestation at delivery	0.57**	0.52**	0.58**	0.34**	0.28***	0.07	0.16†	0.03	0.07	-0.11	0.21**	-0.11	-0.05	0.08	0.09
Infant sex	-0.01	-0.03	0.07	-0.04	-0.14†	0.09	0.00	0.01	0.02	0.03	0.21*	0.10	0.05	-0.13	-0.19*
Age at assessment	--	--	--	--	--	0.05	0.01	-0.03	-0.05	0.01	-0.25**	0.03	0.00	0.10	0.07

[a]Values are Pearson *r*'s.
[b]Age/sex-specific *z*-scores from World Health Organization norms [1]; *n* = 61 controls, 69 heavily exposed at 2 wk; 67 controls, 80 heavily exposed at 5 yr.
[c]From the Fagan Test of Infant Intelligence [2,3]; *n* = 69 controls, 75 heavily exposed at 6.5 mo; 65 controls, 79 heavily exposed at 12 mo.
[d]Highest level of complexity of imitated play [4]; *n* = 64 controls, 78 heavily exposed.
[e]Emotionality, Activity, Sociability Temperament scale [5]; *n* = 70 controls, 81 heavily exposed.
†$p < 0.10$; *$p < 0.05$; **$p < 0.01$; ***$p < 0.001$.

Table S2. Potential interaction effects between prenatal alcohol exposure and iron indices (II) on infant growth, neurobehavior, and temperament[a]

(A) Anthropometry measures

**2-wk length-for-age *z*-scores**[b]	**B_{PAE}**	**B_{II}**	**$B_{PAE\ by\ II}$**	***$P_{PAE\ by\ II}$***
Maternal iron indices				
Ferritin (logged ug/L values)[c]	0.84	-0.24	-0.38	0.273
	(-0.96, 2.63)	(-0.81, 0.34)	(-1.06, 0.30)	
Hemoglobin (g/dL)	-0.88	-0.03	0.05	0.812
	(-5.32, 3.56)	(-0.31, 0.24)	(-0.35, 0.45)	
Hemoglobin:log(ferritin)[d]	-1.90[†]	0.09	0.39	**0.105**
	(-4.02, 0.22)	(-0.27, 0.44)	(-0.08, 0.87)	
Neonatal iron indices				
Ferritin (logged ug/L values)[c]	1.60	0.11	-0.34	0.293
	(-1.78, 4.98)	(-1.78, 4.98)	(-0.97, 0.30)	
Hemoglobin (g/dL)	-1.75	0.06	0.11	0.230
	(-4.43, 0.93)	(-0.05, 0.17)	(-0.07, 0.28)	
Hemoglobin:log(ferritin)[d]	-2.68*	0.02	0.89	**0.021**
	(-4.92, -0.44)	(-0.50, 0.45)	(0.14, 1.65)	

**2-wk weight-for-age *z*-scores**[b]	**B_{PAE}**	**B_{II}**	**$B_{PAE\ by\ II}$**	***$P_{PAE\ by\ II}$***
Maternal iron indices				
Ferritin (logged ug/L values)[c]	0.52	-0.10	-0.31	0.360
	(-1.22, 2.25)	(-0.67, 0.46)	(-0.97, 0.35)	
Hemoglobin (g/dL)	0.22	0.04	-0.06	0.763
	(-3.93, 4.37)	(-0.22, 0.29)	(-0.43, 0.32)	
Hemoglobin:log(ferritin)[d]	-1.29	0.08	0.23	0.323
	(-3.32, 0.73)	(-0.26, 0.42)	(-0.23, 0.68)	
Neonatal iron indices				
Ferritin (logged ug/L values)[c]	0.67	0.06	-0.19	0.541
	(-2.52, 3.85)	(-0.33, 0.45)	(-0.78, 0.41)	
Hemoglobin (g/dL)	-3.21*	0.02	0.20*	**0.020**
	(-5.75, -0.67)	(-0.09, 0.12)	(0.03, 0.36)	
Hemoglobin:log(ferritin)[d]	-2.97**	-0.08	0.96*	**0.011**
	(-5.15, 0.79)	(-0.55, 0.38)	(0.22, 1.70)	

**2-wk head circumference-for-age *z*-scores**[b]	**B_{PAE}**	**B_{II}**	**$B_{PAE\ by\ II}$**	***$P_{PAE\ by\ II}$***

<u>Maternal iron indices</u>				
Ferritin (logged ug/L values)[c]	1.55†	0.15	-0.74*	**0.028**
	(-0.19, 3.28)	(-0.41, 0.71)	(-1.39, 0.08)	
Hemoglobin (g/dL)	1.77	0.14	-0.21	0.291
	(-2.50, 6.05)	(-0.13, 0.41)	(-0.60, 0.18)	
Hemoglobin:log(ferritin)[d]	-2.18*	0.05	0.42†	**0.074**
	(-4.23, -0.13)	(-0.29, 0.40)	(-0.04, 0.88)	
<u>Neonatal iron indices</u>				
Ferritin (logged ug/L values)[c]	-0.76	0.09	0.08	0.803
	(-4.08, 2.57)	(-0.30, 0.49)	(-0.54, 0.70)	
Hemoglobin (g/dL)	-0.68	0.07	0.02	0.799
	(-3.15, 1.79)	(-0.03, 0.17)	(-0.14, 0.18)	
Hemoglobin:log(ferritin)[d]	-0.26	0.13	-0.03	0.943
	(-2.33, 1.80)	(-0.31, 0.56)	(-0.72, 0.67)	

**5-yr length-for-age *z*-scores**[b]	B_{PAE}	B_{II}	$B_{PAE\ by\ II}$	$P_{PAE\ by\ II}$
<u>Maternal iron indices</u>				
Ferritin (logged ug/L values)[c]	-0.19	-0.27	-0.02	0.934
	(-1.48, 1.11)	(-0.69, 0.15)	(-0.51, 0.47)	
Hemoglobin (g/dL)	-3.02†	-0.21	0.25†	**0.081**
	(-6.07, 0.03)	(-0.41, 0.02)	(-0.03, 0.53)	
Hemoglobin:log(ferritin)[d]	-1.31	0.01	0.23	0.176
	(-2.83, 0.22)	(-0.25, 0.28)	(-0.11, 0.57)	
<u>Neonatal iron indices</u>				
Ferritin (logged ug/L values)[c]	1.26	-0.06	-0.30	0.211
	(-1.24, 3.77)	(-0.38, 0.25)	(-0.77, 0.17)	
Hemoglobin (g/dL)	-1.26	0.00	0.06	0.353
	(-3.33, 0.80)	(-0.09, 0.09)	(-0.07, 0.20)	
Hemoglobin:log(ferritin)[d]	-1.95*	0.04	0.59†	**0.059**
	(-3.74, 0.15)	(-0.34, 0.42)	(-0.02, 1.20)	
<u>6.5-mo infant iron indices</u>				
Iron deficiency[c,e]	-0.43*	-0.35	0.42	0.239
	(-0.76, -0.10)	(-0.87, 0.17)	(-0.28, 1.12)	
Iron deficiency anemia[c,e]	-0.11	0.24	-0.47	0.323
	(-0.54, 0.33)	(-0.47, 0.94)	(-1.42, 0.47)	

**5-yr weight-for-age *z*-scores**[b]	B_{PAE}	B_{II}	$B_{PAE\ by\ II}$	$P_{PAE\ by\ II}$
<u>Maternal iron indices</u>				
Ferritin (logged ug/L values)[c]	-0.51	-0.37	0.05	0.838

	(-1.75, 0.73)	(-0.76, 0.03)	(-0.41, 0.52)	
Hemoglobin (g/dL)	-1.18	-0.11	-0.06	0.645
	(-4.16, 1.81)	(-0.30, 0.09)	(-0.21, 0.34)	
Hemoglobin:log(ferritin)[d]	-0.24	0.23†	-0.04	0.803
	(-1.71, 1.23)	(-0.03, 0.48)	(-0.337, 0.28)	
Neonatal iron indices				
Ferritin (logged ug/L values)[c]	1.06	0.11	-0.29	0.227
	(-1.48, 3.60)	(-1.48, 3.60)	(-0.77, 0.19)	
Hemoglobin (g/dL)	-0.51	0.03	0.00	0.987
	(-2.62, 1.59)	(-0.07, 0.12)	(-0.14, 0.14)	
Hemoglobin:log(ferritin)[d]	-1.39	-0.04	0.32	0.316
	(-3.24, 0.46)	(-0.44, 0.35)	(-0.31, 0.95)	
6.5-mo infant iron indices				
Iron deficiency[c,e]	-0.57***	-0.01	0.20	0.560
	(-0.89, 0.25)	(-0.51, 0.49)	(-0.48, 0.88)	
Iron deficiency anemia[c,e]	-0.42†	0.24	-0.31	0.530
	(-0.87, 0.03)	(-0.49, 0.96)	(-1.29, 0.67)	

**5-yr head circumference (cm)**	B_{PAE}	B_{II}	$B_{PAE\ by\ II}$	$P_{PAE\ by\ II}$
Maternal iron indices				
Ferritin (logged ug/L values)[c]	-2.38	-0.05	0.21	0.789
	(-6.47, 1.71)	(-1.36, 1.26)	(-1.34, 1.76)	
Hemoglobin (g/dL)	-6.19	0.08	0.40	0.363
	(-15.63, 3.26)	(-0.53, 0.68)	(-0.46, 1.26)	
Hemoglobin:log(ferritin)[d]	-0.89	0.16	0.21	0.704
	(-5.68, 3.90)	(-0.68, 0.99)	(-1.27, 0.86)	
Neonatal iron indices				
Ferritin (logged ug/L values)[c]	-3.44	0.15	0.32	0.699
	(-12.05, 5.17)	(-0.94, 1.24)	(-1.30, 1.93)	
Hemoglobin (g/dL)	-3.73	0.00	0.13	0.590
	(-10.78, 3.33)	(-0.31, 0.31)	(-0.33, 0.59)	
Hemoglobin:log(ferritin)[d]	-2.30	-0.04	0.17	0.877
	(-8.58, 3.99)	(-1.38, 1.30)	(-1.98, 2.31)	
6.5-mo infant iron indices				
Iron deficiency[c,e]	-1.86***	0.21	0.06	0.954
	(-2.91, -0.82)	(-1.41, 1.84)	(-2.13, 2.25)	
Iron deficiency anemia[c,e]	-1.10***	0.25	-0.16	0.827
	(-1.76, -0.44)	(-0.83, 1.32)	(-1.62, 1.30)	

(B) Neurobehavior

Visual recognition memory (6.5 mo)[f]	B_{PAE}	B_{II}	$B_{PAE\ by\ II}$	$P_{PAE\ by\ II}$
Maternal iron indices				
Ferritin (logged ug/L values)[c]	9.02*	2.26	-3.94*	**0.021**
	(0.09, 17.96)	(-0.52, 5.04)	(-7.28, -0.60)	
Hemoglobin (g/dL)	18.25	0.65	-1.78	0.115
	(-6.20, 42.69)	(-0.78, 2.09)	(-3.99, -0.44)	
Hemoglobin:log(ferritin)[d]	-9.87†	-1.11	-1.92	0.115
	(-20.74, 1.01)	(-2.91, 0.70)	(-0.48, 4.32)	
Neonatal iron indices				
Ferritin (logged ug/L values)[c]	14.68	0.93	2.94	0.123
	(-5.04, 34.41)	(-1.46, 3.32)	(-6.69, 0.81)	
Hemoglobin (g/dL)	9.84	0.52	-0.73	0.170
	(-6.39, 26.08)	(-0.13, 1.16)	(-1.78, 0.32)	
Hemoglobin:log(ferritin)[d]	1.84	1.60	-0.91	0.726
	(-13.46, 17.13)	(-1.34, 4.54)	(-6.02, 4.21)	
6.5-mo infant iron indices				
Iron deficiency[c,e]	-1.09	0.50	-0.30	0.904
	(-3.71, 1.54)	(-3.19, 4.19)	(-5.24, 4.64)	
Iron deficiency anemia[c,e]	-1.95	0.21	-0.03	0.993
	(-5.40, 1.50)	(-4.85, 5.27)	(-6.77, 6.71)	

Visual recognition memory (12 mo)[f]	B_{PAE}	B_{II}	$B_{PAE\ by\ II}$	$P_{PAE\ by\ II}$
Maternal iron indices				
Ferritin (logged ug/L values)[c]	-3.52	0.23	0.41	0.824
	(-13.24, 6.20)	(-2.83, 3.29)	(-3.25, 4.07)	
Hemoglobin (g/dL)	-27.65*	-0.74	2.32*	**0.038**
	(-51.65, -3.66)	(-2.28, 0.81)	(0.13, 4.50)	
Hemoglobin:log(ferritin)[d]	0.68	12.56	-12.87	0.557
	(-9.76, 11.11)	(-23.66, 48.78)	(-56.12, 30.38)	
Neonatal iron indices				
Ferritin (logged ug/L values)[c]	-4.74	-2.13	0.48	0.820
	(-26.66, 17.19)	(-4.82, 0.56)	(-3.66, 4.61)	
Hemoglobin (g/dL)	2.20	-0.30	-0.35	0.531
	(-14.79, 19.20)	(-1.00, 0.41)	(-1.45, 0.75)	
Hemoglobin:log(ferritin)[d]	-11.74	-8.16	23.23	0.268
	(-26.74, 3.25)	(-35.88, 19.57)	(-18.18, 64.64)	

6.5-mo infant iron indices				
Iron deficiency[c,e]	-3.18*	-0.51	2.92	0.275
	(-5.91, -0.46)	(-4.56, 3.54)	(-2.35, 8.19)	
Iron deficiency anemia[c,e]	-4.55*	-3.19	5.77	0.118
	(-8.12, -0.98)	(-8.70, 2.32)	(-1.50, 13.05)	

**Information processing time (6.5 mo)[f]**	B_{PAE}	B_{II}	$B_{PAE\ by\ II}$	$P_{PAE\ by\ II}$
Maternal iron indices				
Ferritin (logged ug/L values)[c]	-0.25	0.02	-0.09	0.437
	(-0.39, 0.89)	(-0.18, 0.22)	(-0.33, 0.14)	
Hemoglobin (g/dL)	1.46	-0.03	-0.13†	**0.091**
	(-0.23, 3.15)	(-0.13, 0.07)	(-0.28, 0.02)	
Hemoglobin:log(ferritin)[d]	0.35	1.27	-1.58	0.274
	(-0.34, 1.04)	(-1.12, 3.67)	(-4.42, 1.26)	
Neonatal iron indices				
Ferritin (logged ug/L values)[c]	0.42	-0.03	0.08	0.447
	(-1.54, 0.70)	(-0.16, -0.11)	(-0.13, 0.29)	
Hemoglobin (g/dL)	-0.06	0.00	0.00	0.997
	(-0.91, 1.03)	(-0.04, 0.04)	(-0.06, 0.06)	
Hemoglobin:log(ferritin)[d]	-0.28	-0.18	0.88	0.450
	(-1.10, 0.54)	(-1.59, 1.23)	(-1.43, 3.19)	
6.5-mo infant iron indices				
Iron deficiency[c,e]	-0.04	-0.07	0.06	0.713
	(-0.22, 0.15)	(-0.33, 0.19)	(-0.28, 0.41)	
Iron deficiency anemia[b,e]	-0.22†	-0.01	0.42†	**0.088**
	(-0.46, 0.02)	(-0.38, 0.35)	(-0.06, 0.91)	

**Information processing time (12 mo)[f]**	B_{PAE}	B_{II}	$B_{PAE\ by\ II}$	$P_{PAE\ by\ II}$
Maternal iron indices				
Ferritin (logged ug/L values)[c]	-0.20	-0.01	0.11	0.356
	(-0.85, 0.44)	(-0.21, 0.19)	(-0.13, 0.35)	
Hemoglobin (g/dL)	0.11	0.01	0.00	0.994
	(-1.51, 1.73)	(-0.09, 0.12)	(-0.15, 0.15)	
Hemoglobin:log(ferritin)[d]	0.50	0.01	-0.09	0.269
	(-0.24, 1.24)	(-0.12, 0.13)	(-0.26, 0.07)	
Neonatal iron indices				
Ferritin (logged ug/L values)[c]	0.50	-0.02	-0.08	0.559

	B_{PAE}	B_{II}	$B_{PAE\ by\ II}$	$P_{PAE\ by\ II}$
	(-0.88, 1.89)	(-0.19, 0.15)	(-0.34, 0.18)	
Hemoglobin (g/dL)	0.20	-0.01	-0.01	0.858
	(-0.89, 1.28)	(-0.08, 0.06)	(-0.08, 0.06)	
Hemoglobin:log(ferritin)[d]	0.04	-0.02	0.02	0.909
	(-0.95, 1.03)	(-0.19, 0.22)	(-0.32, 0.36)	
6.5-mo infant iron indices				
Iron deficiency[c,e]	0.03	0.07	0.22	0.199
	(-0.14, 0.21)	(-0.19, 0.33)	(-0.12, 0.56)	
Iron deficiency anemia[c,e]	0.21	0.10	-0.09	0.725
	(-0.03, 0.45)	(-0.27, 0.46)	(-0.57, 0.40)	

Symbolic Play[g]	B_{PAE}	B_{II}	$B_{PAE\ by\ II}$	$P_{PAE\ by\ II}$
Maternal iron indices				
Ferritin (logged ug/L values)[c]	2.61	0.78	-1.04	0.288
	(-2.47, 7.69)	(-0.88, 2.44)	(-2.97, 0.89)	
Hemoglobin (g/dL)	-5.41	-0.01	0.48	0.392
	(-17.67, 6.85)	(-0.78, 0.76)	(-0.63, 1.60)	
Hemoglobin:log(ferritin)[d]	4.63	-0.62	1.04	0.112
	(-10.46, 1.19)	(-1.65, 0.40)	(-0.25, 2.33)	
Neonatal iron indices				
Ferritin (logged ug/L values)[c]	-1.48	-0.21	0.31	0.744
	(-11.53, 8.58)	(-1.49, 1.07)	(-1.59, 2.21)	
Hemoglobin (g/dL)	-5.43	0.09	0.37	0.161
	(-13.43, 2.57)	(-0.27, 0.44)	(-0.15, 0.90)	
Hemoglobin:log(ferritin)[d]	3.46	-0.47	1.28	0.306
	(-10.71, 3.79)	(-10.71, 3.79)	(-1.19, 3.75)	
6.5-mo infant iron indices				
Iron deficiency[c,e]	-0.30	-0.30	-0.66	0.625
	(-2.35, 1.75)	(-2.35, 1.75)	(-3.33, 2.01)	
Iron deficiency anemia[c,e]	0.18	-0.01	-1.92	0.251
	(-1.03, 1.39)	(-2.57, 2.54)	(-5.20, 1.37)	

(C) Temperament[h]

Emotionality	B_{PAE}	B_{II}	$B_{PAE\ by\ II}$	$P_{PAE\ by\ II}$
Maternal iron indices				
Ferritin (logged ug/L values)[c]	0.24	0.08	-0.06	0.603
	(-0.34, 0.82)	(-0.11, 0.26)	(-0.27, 0.16)	

Hemoglobin (g/dL)	1.19	0.07	-0.10	0.143
	(-0.27, 2.65)	(-0.02, 0.16)	(-0.23, 0.03)	
Hemoglobin:log(ferritin)[d]	0.01	0.13	0.37	0.776
	(-0.61, 0.63)	(-2.05, 2.31)	(-2.19, 2.92)	
Neonatal iron indices				
Ferritin (logged ug/L values)[c]	-0.71	0.15*	-0.12	0.276
	(-0.48, 1.90)	(0.00, 0.29)	(-0.35, 0.10)	
Hemoglobin (g/dL)	0.57	0.01	-0.03	0.346
	(-0.45, 1.59)	(-0.04, 0.05)	(-0.10, 0.03)	
Hemoglobin:log(ferritin)[d]	0.19	0.85	-0.35	0.779
	(-0.69, 1.07)	(-0.84, 2.55)	(-2.82, 2.12)	
6.5-mo infant iron indices				
Iron deficiency[c,e]	0.10	0.12	0.05	0.740
	(-0.07, 0.26)	(-0.12, 0.35)	(-0.26, 0.36)	
Iron deficiency anemia[c,e]	0.11	0.03	0.00	0.998
	(-0.10, 0.32)	(-0.30, 0.36)	(-0.43, 0.43)	

**Activity**	B_{PAE}	B_{II}	$B_{PAE\ by\ II}$	$P_{PAE\ by\ II}$
Maternal iron indices				
Ferritin (logged ug/L values)[c]	0.07	0.06	-0.05	0.653
	(-0.57, 0.71)	(-0.14, 0.26)	(-0.25, 0.18)	
Hemoglobin (g/dL)	-0.10	0.03	0.00	0.973
	(-1.72, 1.53)	(-0.07, 0.13)	(-0.15, 0.15	
Hemoglobin:log(ferritin)[d]	0.25	-0.02	0.04	0.508
	(-0.98, 0.49)	(-0.15, 0.11)	(-0.12, 0.21)	
Neonatal iron indices				
Ferritin (logged ug/L values)[c]	0.11	0.08	-0.03	0.798
	(-1.27, 1.49)	(-0.10, 0.25)	(-0.29, 0.23)	
Hemoglobin (g/dL)	-0.84	-0.01	0.05	0.154
	(-1.96, 0.28)	(-0.06, 0.04)	(-0.02, 0.13)	
Hemoglobin:log(ferritin)[d]	-0.82	-0.13	-0.27	0.120
	(-1.83, 0.19)	(-0.34, 0.80)	(-0.07, 0.61)	
6.5-mo infant iron indices				
Iron deficiency[c,e]	-0.05	-0.26*	0.07	0.673
	(-0.22, 0.13)	(-0.52, 0.00)	(-0.26, 0.41)	
Iron deficiency anemia[c,e]	-0.04	-0.29	0.13	0.574
	(-0.25, 0.18)	(-0.64, 0.06)	(-0.32, 0.58)	

Sociability	B_{PAE}	B_{II}	$B_{PAE by II}$	$P_{PAE by II}$
Maternal iron indices				
Ferritin (logged ug/L values)[c]	0.15	-0.03	-0.04	0.721
	(-0.48, 0.79)	(-0.23, 0.17)	(-0.28, 0.20)	
Hemoglobin (g/dL)	0.32	-0.08	-0.03	0.726
	(-1.28, 1.92)	(-0.18, 0.03)	(-0.17, 0.12)	
Hemoglobin:log(ferritin)[d]	-0.17	-0.01	0.05	0.595
	(-0.92, 0.58)	(-0.14, 0.12)	(-0.12, 0.21)	
Neonatal iron indices				
Ferritin (logged ug/L values)[c]	0.70	-0.02	-0.13	0. 354
	(-0.76, 2.17)	(-0.20, 0.17)	(-0.41, 0.15)	
Hemoglobin (g/dL)	0.46	0.01	-0.03	0.438
	(-0.74, 1.67)	(-0.04, 0.06)	(-0.11, 0.05)	
Hemoglobin:log(ferritin)[d]	0.20	0.10	-0.07	0.721
	(-0.89, 1.29)	(-0.14, 0.33)	(-0.43, 0.30)	
6.5-mo infant iron indices				
Iron deficiency[c,e]	0.02	-0.11	-0.02	0.922
	(-0.16, 0.21)	(-0.37, 0.16)	(-0.36, 0.33)	
Iron deficiency anemia[c,e]	-0.13	-0.18	0.32	0.200
	(-0.37, 0.12)	(-0.56, 0.19)	(-0.17, 0.80)	

Shyness	B_{PAE}	B_{II}	$B_{PAE by II}$	$P_{PAE by II}$
Maternal iron indices				
Ferritin (logged ug/L values)[c]	-0.26	-0.09	0.13	0.319
	(-0.97, 0.45)	(-0.31, 0.14)	(-0.13, 0.40)	
Hemoglobin (g/dL)	0.20	0.09	-0.01	0.885
	(-1.60, 1.99)	(-0.03, 0.20)	(-0.18, 0.15)	
Hemoglobin:log(ferritin)[d]	-0.39	-1.87	2.10	0.186
	(-1.15, 0.36)	(-4.52, 0.78)	(-1.02, 5.21)	
Neonatal iron indices				
Ferritin (logged ug/L values)[c]	-0.78	-0.08	0.17	0.247
	(-2.30, 0.74)	(-0.27, 0.11)	(-0.12, 0.46)	
Hemoglobin (g/dL)	-0.24	-0.03	0.02	0.604
	(-1.52, 1.05)	(-0.08, 0.03)	(-0.06, 0.11)	
Hemoglobin:log(ferritin)[d]	-0.11	-0.24	0.52	0.742
	(-1.23, 1.02)	(-1.92, 2.39)	(-2.61, 3.65)	
6.5-mo infant iron indices				

Iron deficiency[c,e]	0.12<br>(-0.07, 0.32)	0.28*<br>(0.00, 0.57)	-0.19<br>(-0.56, 0.19)	0.334
Iron deficiency anemia[c,e]	0.09<br>(-0.18, 0.35)	0.15<br>(-0.26, 0.56)	-0.24<br>(-0.77, 0.29)	0.369

[a]Values are raw regression coefficients (95% confidence intervals) from linear regression models regressing a given outcome on PAE (B_{PAE}), the given iron measure (B_{II}), a PAE x iron measure outcome ($B_{PAE\ by\ II}$, $p_{PAE\ by\ II}$), and potential confounders (maternal age, prenatal cigarettes/day, maternal education, and wk gestation at delivery for anthropometry measures, with the addition of age at time of measurement for 5-yr head circumference; maternal age, gravidity, and wk gestation at delivery for visual recognition memory; maternal education, wk gestation at delivery, infant sex, and age at time of assessment for symbolic play; gravidity and socioeconomic status for emotionality; infant sex for activity; gravidity and prenatal methamphetamine use (days/mo) for sociability; prenatal cigarette smoking (cigarettes/day) and marijuana use (days/mo) for shyness).

[b]Age/sex-specific *z*-scores from World Health Organization norms [1]; *n* = 61 controls, 69 heavily exposed at 2 wk; 67 controls, 80 heavily exposed at 5 yr.

[c]Positively related to prenatal alcohol exposure in previously published analyses [6].

[d]Negatively related to prenatal alcohol exposure in previously published analyses [6].

[e]Yes = 1; no = 0.

[f]From the Fagan Test of Infant Intelligence [2,3]; *n* = 69 controls, 75 heavily exposed at 6.5 mo.

[g]Highest level of complexity of imitated play [4]; *n* = 64 controls, 78 heavily exposed.

[h]Emotionality, Activity, Sociability Temperament scale [5]; *n* = 70 controls, 81 heavily exposed.

†$p < 0.10$; *$p < 0.05$; **$p < 0.01$; ***$p < 0.001$.

References

1. de Onis, M.; Garza, C.; Victora, C.G.; Onyango, A.W.; Frongillo, E.A.; Martines, J. The WHO Multicentre Growth Reference Study: planning, study design, and methodology. *Food Nutr Bull* **2004**, *25*, S15-26.
2. Fagan, J.F.; Singer, L.T. Infant recognition memory as a measure of intelligence. In *Advances in infancy research*, Lipsett, L.P., Ed. Ablex: Norwood, NJ, 1983; Vol. 2.
3. Jacobson, S.W.; Jacobson, J.L.; O'Neill, J.M.; Padgett, R.J.; Frankowski, J.J.; Bihun, J.T. Visual expectation and dimensions of infant information processing. *Child Dev* **1992**, *63*, 711-724.
4. Belsky, J.; Garduque, L.; Hrncir, E. Assessing performance, competence, and executive capacity in infant play. *Dev Psychol* **1984**, *20*, 406-417.
5. Buss, A.H.; Plomin, R. *Temperament: Early Developing Personality Traits*; Lawrence Erlbaum: Hillsdale, NJ, 1984.
6. Carter, R.C.; Georgieff, M.K.; Ennis, K.M.; Dodge, N.C.; Wainwright, H.; Meintjes, E.M.; Duggan, C.P.; Molteno, C.D.; Jacobson, J.L.; Jacobson, S.W. Prenatal alcohol-related alterations in maternal, placental, neonatal, and infant iron homeostasis. *Am J Clin Nutr* **2021**, *114*, 1107-1122, doi:10.1093/ajcn/nqab165.
